# Data-Driven Chance Constrained Mixed Integer Nonlinear Bilevel Optimization via Copulas

**DOI:** 10.1021/acs.iecr.5c04768

**Published:** 2026-04-21

**Authors:** Syu-Ning Johnn, Hasan Nikkhah, Meng-Lin Tsai, Styliani Avraamidou, Burcu Beykal, Vassilis M. Charitopoulos

**Affiliations:** † Department of Chemical Engineering, The Sargent Centre for Process Systems Engineering, 4919 University College London, London WC1E 7JE, U.K.; ‡ Department of Chemical & Biomolecular Engineering, 7712 University of Connecticut, Storrs, Connecticut 06269, United States; § Center for Clean Energy Engineering, 5228 University of Connecticut, Storrs, Connecticut 06269, United States; ∥ Department of Chemical & Biological Engineering, University of Wisconsin-Madison, Madison, Wisconsin 53706, United States

## Abstract

Process supply chains have at their core the functionalities of planning and scheduling as means to ensure their profitability and responsive operations. At the same time, challenges in real-world demand data, such as multivariate data dependency and correlations, noisy data distributions, and parameter uncertainties, significantly complicate the decision-making process, making it difficult to identify optimal solutions. This necessitates the adoption of data-driven optimization approaches to effectively account for the dependency structures inherent in the observed data, thereby efficiently exploring solution spaces and identifying high-quality outcomes. This work proposes a data-driven probabilistic framework that integrates chance constrained programming (CCP) and copulas. CCP is an optimization-based method that enforces probabilistic constraints under specified risk thresholds. Copulas are a statistical technique designed to estimate data while capturing the dependency structure among multiple uncertain parameters with inherent correlations. The proposed framework is capable of accurately modeling variable dependencies across different scenarios, especially when the data exhibit complex distributions or nontrivial dependencies. We focus on three integrated case studies originating from the planning and scheduling of process supply chains and crude oil scheduling. We combine our proposed estimation framework with the Data-driven Optimization of bilevel Mixed-Integer NOnlinear problems (DOMINO) framework, which is a data-driven gray-box algorithm for addressing bilevel optimization problems, to derive decisions with guaranteed demand satisfaction rates. Computational experiments demonstrate that our proposed copula-based chance constrained optimization framework can incorporate demand correlation and achieve a higher joint demand satisfaction rate, lower total costs, and higher efficiency.

## Graphical abstract

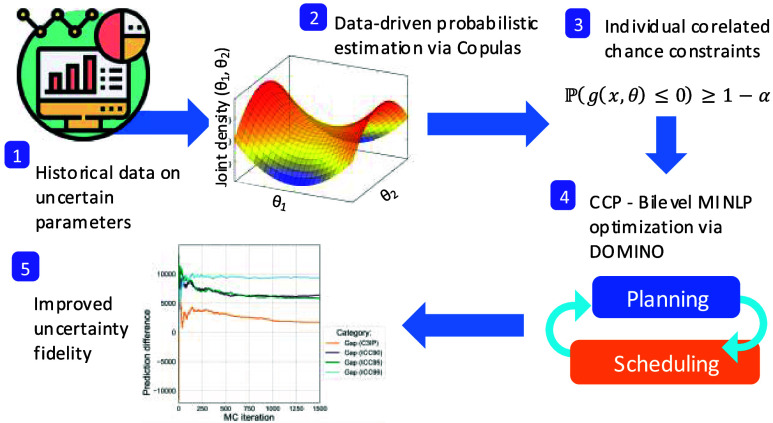

## 1 Introduction

Amid the rise of big data, the rapid growth in data volumes presents promising opportunities for advanced, data-driven decision-making in real-world industrial applications. Traditional deterministic methods rely on oversimplified models that are often insufficient in capturing uncertainty, nonlinear dynamics, and strong system interdependencies, thereby limiting their effectiveness in leveraging data resources. As a result, finding optimal or even feasible solutions for many industrially relevant optimization problems remains intrinsically challenging. Several key obstacles contribute to this difficulty, including the lack of prior knowledge regarding data distributions, the presence of stochastic demand fluctuations, inner-data correlations and structures, and parameter uncertainties, all of which hinder the ability of traditional models to effectively interpret complex problem structures and identify high-quality solutions to support a refined decision-making process. In contrast, data-driven optimization (DDO) leverages the granularity of real-world data, offering a distinct advantage in extracting intricate data patterns to enhance decision-making.^47^ Consequently, DDO has been increasingly adopted to identify data value, utilizing statistical resources to support smarter operations. One such class of problems and of interest to this work is the enterprise wide optimization paradigm, nominally introduced by Grossmann.^22^ Uncertainty is a key practical factor in process operations, which has been the focus of substantial research attention in many fields, including microgrid market bidding,^23^ online scheduling,^27^ stochastic industrial gas market modeling^32^ and hydrogen infrastructure planning.^50^ Specifically, the integration of planning and scheduling has attracted the interest of research community over the last two decades owing to the benefits that decision-makers can gain by exploiting the underlying interdependence of the aforementioned functionalities.^18,25,31^ To this end, different frameworks have been proposed to capture this interdependence but at the same time balance the computing effort resulting from the multiscale nature of the integrated problem.^43^

Chance constrained programming (CCP) is an optimization approach for decision-making in the presence of uncertainties that guarantees stochastic constraints are satisfied with a certain reliability level, thus offering controlled flexibility to manage risk and account for the inherent variabilities in decision processes. Numerous studies have successfully integrated CCP within various optimization problems,^20^ with applications in energy production,^49^ airport operation management,^46^ water resources management,^2^ supply chain management,^35^ power system optimization,^49^ stochastic project network scheduling,^8^ and financial risk management.^15^

To ensure the reliability of CCP, a statistical method is required to model and evaluate the dependence structure among multiple uncertain parameters. Copulas are a class of data-driven coupling functions specifically designed to capture the characteristics of dependency among multiple random variables with inherent correlations. Integrating copula formulations into the CCP framework enhances its ability to better model dependencies between variables under different scenarios and leverage historical data patterns, particularly when the underlying data exhibits complex distributions, nontrivial dependencies, and correlated risks. This integration leads to improved estimation accuracy during the decision-making process in optimization problems in the presence of uncertain parameters.

In recent years, the integration of copulas and CCP has shown significant promise, utilizing the strengths of both to develop risk-based frameworks enabling quantitative analysis regarding different sources of uncertainty and their dependencies. Ding et al.^19^ developed a copulas-based CCP framework that utilizes vine copulas to conduct risk management, focusing on multidimensional correlated uncertainties related to management efficiencies, aimed at addressing nonpoint source pollution for water quality management. Khezri and Khodayifar^28^ applied joint chance constraints to address the capacity requirement in a multicommodity network flow problem, using an Archimedean copula to estimate the dependencies between different capacity coefficient variables. Hosseini Nodeh et al.^24^ employed copulas to model the dependency between resource consumptions of a stochastic resource-constrained shortest path problem. Alizadeh et al.^1^ combined copulas and CCP to address multiple uncertain sources emerging from the water reservoir irrigation district systems optimization, aiming to balance the interrelationships among optimal values of the agro-economic parameters, the target irrigation supply reliability levels, and the decision-maker’s risk attitude. Chen et al.^16^ proposed a joint chance constrained method that integrates the best-performing copula from a set of widely applied bivariate copulas to characterize the uncertain probability distributions and the correlated nature of multiperiod, multizone residential waste data.

In this work, we propose a data-driven chance constrained framework designed to transform uncertainties to quantitative risk levels. This framework incorporates integrated copulas to estimate multivariate dependent sources of uncertainties within the context of the process scheduling optimization problem. The chance constrained-based approach offers clear advantages over the traditional scenario-based method, as it eliminates the need to explicitly track every scenario, thereby reducing resource intensity and computational time requirements. Moreover, we integrate our proposed optimization framework with the DOMINO (Data-driven Optimization of bilevel Mixed-Integer NOlinear problems) framework,^5^ which is a data-driven gray-box algorithm designed to address bilevel optimization problems with various formulations at the lower levels.^7^ The DOMINO framework has previously been shown to address both deterministic^6,39,40^ and stochastic^7^ integrated planning and scheduling problems, with the latter handled through scenario-based optimization. The goal of this work is to reduce the computational burden of solving repeated scenarios in DOMINO while guaranteeing demand satisfaction via a copula-based chance constrained optimization approach.

The novelty of this work is the combination of chance constraints and DOMINO to form an efficient data-driven optimization method that preserves dependency structure via copulas technique. Specifically, the integration of chance constrained copula into the DOMINO framework yields a more efficient risk-based decision-making process, in which uncertainty parameters are estimated based on predefined risk levels and inner-data dependencies rather than through explicit computation of all scenarios, thereby substantially reducing computational time relative to existing stochastic programming-based frameworks in.^7^

We test our optimization framework on a motivating single-stage continuous stirred-tank reactor and a higher-dimensional multiproduct crude oil planning and scheduling case study. Our results show that the proposed copulas-based data-driven chance constrained model demonstrates superior capability in producing robust decisions with higher profits and lower penalties compared to the traditional methods with similar risk levels. The computational experiments further demonstrate that the proposed chance constrained copula method effectively identifies robust solutions, yielding higher joint satisfaction rates for products and near-optimal performance, while also significantly reducing computational time relative to existing stochastic optimization methods.

The remainder of the paper is organized as follows. Section 2 introduces the copula theory, chance constrained formulation, and the integration of the two to form the data-driven chance constrained copula framework. Section 3 presents our proposed data-driven chance constrained copula framework through a motivating study and two case studies. Conclusions and future work are included in Section 4.

## 2 Methodology

### 2.1 Copula Theory

Copulas are essential statistical tools for comprehensively analyzing the dependency structures exhibited in multivariate data distributions. Copulas can be categorized into parametric, semiparametric, or nonparametric, allowing flexible specification of marginal distributions. An *n*-dimensional copula function *C* joins or “couples” multiple marginal cumulative distributions from unknown underlying distributions to form a multivariate joint distribution on [0, 1]^*n*^ while preserving the interdistribution dependency structure. Sklar’s Theorem^42^ formalizes the mathematical foundation that any multivariate distribution function *F*(*x*_1_, *x*_2_,···, *x*_*n*_) can be formulated in terms of two distinct components: a set of univariate marginal distribution functions *F*_*i*_(*x*_*i*_) with *i* ∈ {1,···, *n*}, each function representing the individual variable’s behavior, together with the predetermined *n*-dimensional copula function *C*, which encapsulates the dependency structure between the marginal distributions as shown by eq 1.

$$F\left(x_{1},x_{2},...,x_{n}\right)=C\left(F_{1}\left(x_{1}\right),F_{2}\left(x_{2}\right),...,F_{n}\left(x_{n}\right)\right)$$ 1

where *x*_1_, *x*_2_, ..., *x*_*n*_ are the realizations of the random variables (*x*) from the marginal distributions *F*_1_, *F*_2_,···,*F*_*n*_. This enables the construction of any multivariate distribution using a chosen copula and specified marginal distributions based on observed data, regardless of whether the underlying real distribution is fully defined. Copula functions are uniquely defined in the case when the underlying marginal distributions are continuous. A few commonly applied copula models include Gaussian copulas,^33^ t-copulas,^11^ and Archimedean copulas,^21^ each tailored to specific types of dependency structures.

A copula function is termed as bivariate when two random variables are involved (*n* = 2). Given the calculation of the probability density function $f\left(x\right)=\frac{\partial F\left(x\right)}{\partial x}$ using derivatives, a bivariate copula captures the pairwise dependency structure between the two variables, each of which is associated with a marginal distribution. We can therefore derive the joint density function using the copula density function *c*_12_ and the two marginal density functions as given below by eq 2.

2 f(x1,x2)=∂2F(x1,x2)∂x1∂x2=∂2C12(F1(x1),F2(x2))∂x1∂x2=c12(F1(x1),F2(x2))·f1(x1)·f2(x2)

In other words, any bivariate copula density function can be derived using marginal and joint density functions of the two underlying random variables. We can therefore generalize eq 2 into higher dimensional with multiple variables and associated marginal distributions as shown by eq 3.

$$f\left(x_{1},x_{2},...,x_{n}\right)=c\left(F_{1}\left(x_{1}\right),F_{2}\left(x_{2}\right),...,F_{n}\left(x_{n}\right)\right)\cdot f_{1}\left(x_{1}\right)...f_{n}\left(x_{n}\right)$$ 3

A vine copula is a construction method that decomposes a high-dimensional, multivariate dependency structure (*n* ≥ 3) into a sequence of pairwise bivariate copulas and marginal density functions. Given the conditional density function *f*(*x*_1_|*x*_2_) = $\frac{f\left(x_{1},x_{2}\right)}{f\left(x_{2}\right)}$ , we can construct a higher-dimensional vine copula function containing multiple variables, based on univariate conditional density functions and bivariate copulas. Assuming that three variables are included in the example applying the conditional density function eq 4,

4 $$f\left(x_{1},x_{2},x_{3}\right)=f\left(x_{1}|x_{2},x_{3}\right)\cdot f\left(x_{2}|x_{3}\right)\cdot f\left(x_{3}\right)$$

where the conditional terms can be further derived via eqs 5 and 6.

$$f\left(x_{1}|x_{2},x_{3}\right)=c_{1,2|3}\left(F_{1|3}\left(x_{1}|x_{3}\right),F_{2|3}\left(x_{2}|x_{3}\right)\right)\cdot f\left(x_{1}|x_{3}\right)=c_{1,2|3}\left(F_{1|3}\left(x_{1}|x_{3}\right),F_{2|3}\left(x_{2}|x_{3}\right)\right)\cdot c_{1,3}\left(F_{1}\left(x_{1}\right),F_{3}\left(x_{3}\right)\right)\cdot f\left(x_{1}\right)$$ 5

$$f\left(x_{2}|x_{3}\right)=c_{2,3}\left(F_{2}\left(x_{2}\right),F_{3}\left(x_{3}\right)\right)\cdot f\left(x_{2}\right)$$ 6

Given that the copula density corresponding to the distribution of (*X*_1_, *X*_2_) conditional to *X*_3_ = *x*_3_ is denoted by *c*_1,2|3_, we can derive the joint density function via bivariate copulas and marginal distribution as shown by eq 7.

7 $$f\left(x_{1},x_{2},x_{3}\right)=f\left(x_{1}\right)\cdot f\left(x_{2}\right)\cdot f\left(x_{3}\right)\cdot c_{1,3}\left(F_{1}\left(x_{1}\right),F_{3}\left(x_{3}\right)\right)\cdot c_{2,3}\left(F_{2}\left(x_{2}\right),F_{3}\left(x_{3}\right)\right)\cdot c_{1,2|3}\left(F_{1|3}\left(x_{1}|x_{3}\right),F_{2|3}\left(x_{2}|x_{3}\right)\right)$$

By focusing on the joint distribution of marginal quantiles, vine copulas preserve the underlying dependencies among variables while allowing us to abstract from individual marginal characteristics. Since the choice of which variables to condition upon directly determines which set of bivariate copulas to be applied inside eq 7 and consequently the underlying dependency structure among the variables. Therefore, there is enormous flexibility with the copula structure if the order of variables is shifted inside the multivariate function (e.g., assuming *f*(*x*_1_, *x*_3_, *x*_2_) or *f*(*x*_3_, *x*_2_, *x*_1_) as the function to be decomposed). The enumeration of possible combinations influences which pairs of variables should be directly coupled, the selection of conditional functions and the particular bivariate copula functions, with an increasing impact especially when generalized to higher dimensions. Consequently, the choice of variable order plays a critical role in determining the conditional relationships to discover strong correlations within data in order to maximize dependency, and constitutes one of the key hyperparameters of the method.

Compared to fitting a single multivariate copula to the high-dimensional data set, a vine copula is built out of bivariate copulas that breaks the joint distribution to estimate complex correlations with better flexibility and tractability as the number of variables grows. Moreover, unlike other multivariate copulas that assume symmetric or elliptical dependence, vine copulas offer better capability to capture complex, asymmetries, nonlinear, and tail dependencies (extreme events) that traditional correlation metrics may often overlook.^9^ In this work, we employ vine copulas to determine the copula family and structure, thereby maximizing dependency and maintaining strong intervariable correlations.

### 2.2 Chance Constrained Programming

Chance Constrained Programming (CCP) is an optimization-based technique that formulates probabilistic constraints to model uncertainty in decision-making problems.^30^ This approach addresses uncertainty by allowing probabilistic constraint(s) to be satisfied with a predetermined reliability level.^10^

An individual probabilistic constraint, known as an individual chance constraint (ICC), specifies that the constraint must be satisfied with a certain confidence level, denoted as a probability term (1−α)%, where α represents the risk level for the given constraint.^10^ This probabilistic interpretation enables a controlled trade-off between strict feasibility and a degree of flexibility in constraint compliance, which is particularly useful in dynamic or uncertain environments. For instance, assigning a risk level of α = 0.05 indicates that the constraint is expected to hold with at least 95% probability while permitting a 5% risk tolerance for potential violation of the particular constraint. The most general form of an ICC is given below as eq 8. Each ICC can also be equivalently expressed in terms of inverse cumulative distribution function (CDF) of the uncertainty as eq 9 as follows.

8a $$P\left(g_{i}\left(x,\xi \right)\leq 0\right)\geq 1-\alpha_{i},\forall i\in I$$

8b $$\inf\left\{z:P\left(g_{i}\left(x,\xi \right)\leq z\right)\geq 1-\alpha_{i}\right\}\leq 0,\forall i\in I$$

where in the general form eq 8, the decision vector *x* and random vector ξ are within the constraint function *g*(*x*, ξ), together with the violation probability α, which show that each constraint must hold with its own probability level, such that the specified demand requirement constraint is fulfilled with a probability no less than (1−α_*i*_)% for each individual scenario or realization. Each of the reformulated inverse CDF constraint, equivalently a quantile constraint, enforces that 1−α_*i*_-quantile of the of the random variable does not exceed zero, thereby ensuring the original probabilistic requirement.

Joint chance constraint (JCC) is a variation of ICC, distinguished by its emphasis on simultaneously accounting for multiple constraints with a single given confidence level. Similarly, the JCC formulations can be represented mathematically in general form eq 10 and reformulated as an inverse CDF (quantile) constraint eq 11 as follows:

9a $$P\left(g_{i}\left(x,\xi \right)\leq 0,\forall i\in I\right)\geq 1-\beta$$

9b $$\inf\left\{z\in R:P\left(\max_{i\in I}g_{i}\left(x,\xi \right)\leq z\right)\geq 1-\beta \right\}\leq 0$$

where the inverse CDF reformulation eq 11 specifies that 1−β-quantile of the maximum constraint violation random variable must be less than or equal to 0. Consequently, the JCC approach imposes tighter constraints and hence stricter limitations on the feasible region of the solution space, making the resulting optimization problem generally more challenging to solve in practice.

In general, CCP offers a simplified uncertainty estimation approach based on risk level, which is more efficient compared to alternative methods such as stochastic programming, as it requires explicit representation of all scenarios and incurs a computational burden for high-dimensional problems.

In this work, we employ copula to capture the dependency among ICC entities, enforcing constraints at their individual risk levels. The reason for choosing ICC over JCC is that the selection of ICC enhances flexibility by allowing different demands to follow different uncertainty levels. By decomposing into marginal or lower-dimensional probabilities, ICC avoids the need to compute a high-dimensional nonlinear joint probability. Consequently, the optimization process is more computationally tractable, while their correlated dependence behaviors are still preserved via copulas.

### 2.3 Copula-based Data-driven Chance Constrained Programming

Formulating and solving chance constrained problems is inherently challenging due to the uncertainty surrounding the probability distribution of historical data. The distributions are often unknown or difficult to model accurately when only limited observed data is available. Estimating an unknown distribution based on existing parametric distributions (e.g., the Gaussian distribution) becomes challenging in high-dimensional or nonlinear contexts with no closed-form formulations, which often necessitates approximation or data-driven empirical methods to generate estimators that can more accurately capture the actual distribution. This challenge is further compounded when correlation is taken into account, as it necessitates a concurrent consideration of dependency structure among the marginal probability distributions to ensure accurate modeling of the unknown distribution.

We propose Copulas-based Individual Chance Constrained Programming (**C** ^**3**^ **IP**), a data-driven framework that integrates copulas into data-driven chance constrained optimization to model dependencies between variables, as illustrated in Figure 1.

**Figure 1:**
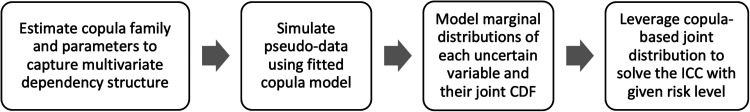
Logic flow for the proposed data-driven C^3^IP framework with copulas-estimated correlated scenarios.

The integration of the copulas technique into the modeling of C^3^IP provides a data-driven approach based on empirical data. This way, we leverage both the marginal distributions of uncertain variables and their dependency structures to construct joint distributions that account for correlated dynamics among individual distributions. The framework begins with fitting the copulas to data using *pyvinecopulib*,^38^ a Python interface that covers a class of dependence models with bivariate copula building blocks to model and simulate complex multivariate dependencies. During the first step, maximum likelihood estimation was applied to identify the most compatible dependency structure and estimate the best-fitting copula family as well as the associated parameters. The idea is to capture strong pairwise bivariate copula dependencies to maximize overall dependency in the employed vine copula. Subsequently, we construct a surrogate model based on the fitted copula algorithm, which simulates correlated pseudo-observations. Upon completion of the simulated copula fitting, synthetic samples are generated from the fitted copula model. The fitted copula generates pseudo-observations, which are derived from the sample data and transformed into a uniform distribution while retaining the rank order of the original data, thus preserving the original dependency structure between variables. The transformation process offers enhanced scalability and robustness by reconstructing complete data sets with inferred dependency structure while stripping away the marginal distributions. Afterward, the marginal distributions can be estimated using nonparametric methods to unknown true continuous probability distribution functions based on empirical data. In our approach, we adapt the built-in function in *pyvinecopulib* to infer the joint CDF based on underlying marginal distributions. Finally, once the vine copula and marginal distributions are fitted, the joint distribution can be built via eq 1 by combining the fitted copula and the marginals. It can subsequently be utilized to approximate both the quantile function (inverse CDF) with the associated risk level. Algorithm 1 provides a summary of the steps employed.

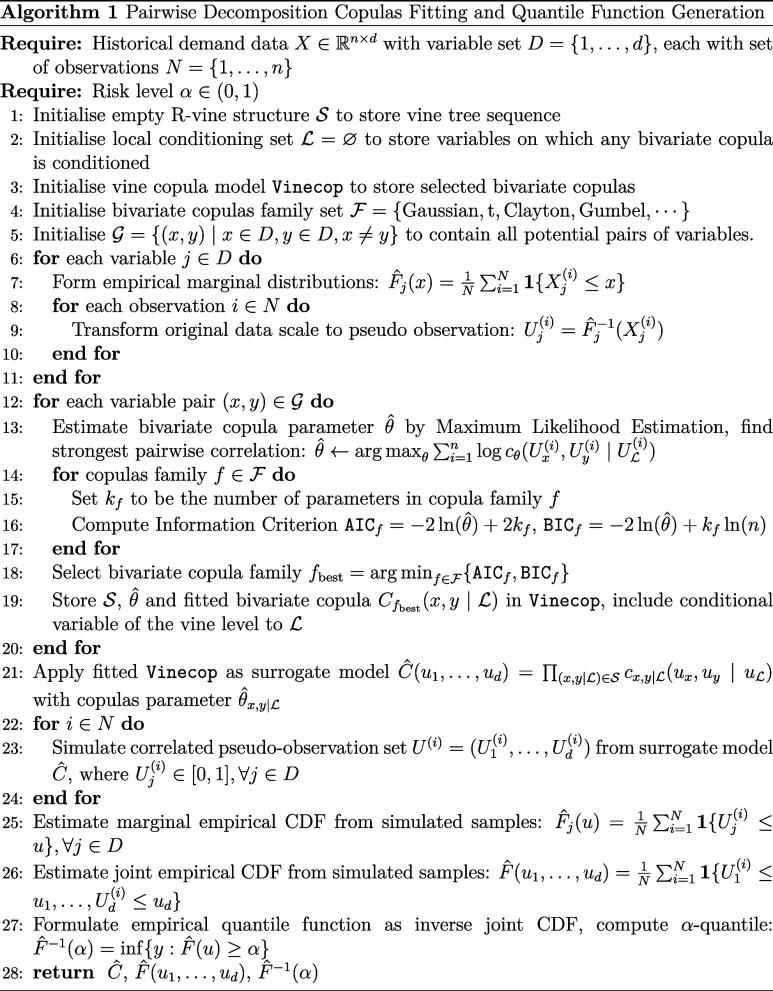



Building on the data-driven copula fitting and simulation process outlined above, we evaluate the performance of through a Monte Carlo (MC) simulation. In this process, each scenario generated from the copula-based quantile function serves as input to the problem instance, allowing for the computation of the probability of constraint satisfaction across all realizations a posteriori.

### 2.4 Data-Driven Mixed Integer Nonlinear Bilevel Optimization via DOMINO

The proposed C^3^IP-DOMINO framework constitutes a generalized solution methodology for bilevel optimization problems characterized by a hierarchical decision structure, where the upper-level problem contains another lower-level optimization problem as part of its constraints. In the context of bilevel integrated planning and scheduling (iPS) problem, the historical demand data is utilized to compute the copula-estimated demand set associated with a particular risk level following the procedure shown in Figure 1. The generated demand set from C^3^IP model can then be utilized as a demand scenario of iPS optimization problems^12,14,17^ to be solved using the DOMINO framework. DOMINO^5^ is a data-driven framework for solving general constrained bilevel problems,^36,37,45^ a topic that has generated significant research interest.^3,41,48^

Here, we briefly outline how DOMINO handles such problems and the specific roles of the solvers involved in the optimization process, using two specific iPS problems in the context of a planning and scheduling manufacturing process introduced in^12^ and a crude oil refinery process introduced in.^40^ The mathematical form of the bilevel iPS problem can be found in Appendix A. The framework operates by sampling upper-level decision variables (e.g., planning targets), solving the corresponding lower-level problems (e.g., scheduling) to global optimality using a deterministic solver such as BARON,^44^ and then using these evaluations to build a gray-box optimization model. A data-driven optimizersuch as the local search algorithm NOMAD (Nonlinear Optimization by Mesh Adaptive Direct Search)^29^ or a global algorithm is then used to search for optimal upper-level decisions. This cycle of sampling, lower-level optimization, and data-driven upper-level optimization continues iteratively. The process continues until convergence criteria are satisfied, which include reaching the maximum number of samples, a lack of improvement over several iterations, or CPU time limit. For a comprehensive description of the DOMINO framework, we refer the reader to.^5^ Notice that we use data-driven optimization as the solution strategy, which like other data-driven optimization method in this class, does not guarantee that a feasible solution will always be identified within an empirical data set with finite computational budget Nonetheless, an important distinction is that whenever a feasible solution is found, its feasibility is guaranteed by the DOMINO solver. In this sense, while global discovery cannot be guaranteed a priori, any reported solution is provably feasible.

Figure 2 illustrates the workflow between the copulas and DOMINO that forms the C^3^IP-DOMINO framework. The upper subplot depicts the conventional ICC model, where uncertainty is characterized by individual risk thresholds under the assumption that all demands are independent and uncorrelated. In contrast, the lower subplot presents the C^3^IP-DOMINO model, in which parameter correlations are estimated via copulas. All scenarios are first generated under multivariate normal distribution and then partitioned into two groups: a training group and an evaluation group. The training group is used to fit copulas, which capture the joint distribution of the relevant variables. Once the copula model is trained and the distribution has been estimated, quantile estimation is applied to reformulate the chance constraints. The evaluation group, which is independent of the training set, is subsequently used in the case studies, specifically Monte Carlo simulations, to assess and showcase the performance of the proposed framework.

**Figure 2:**
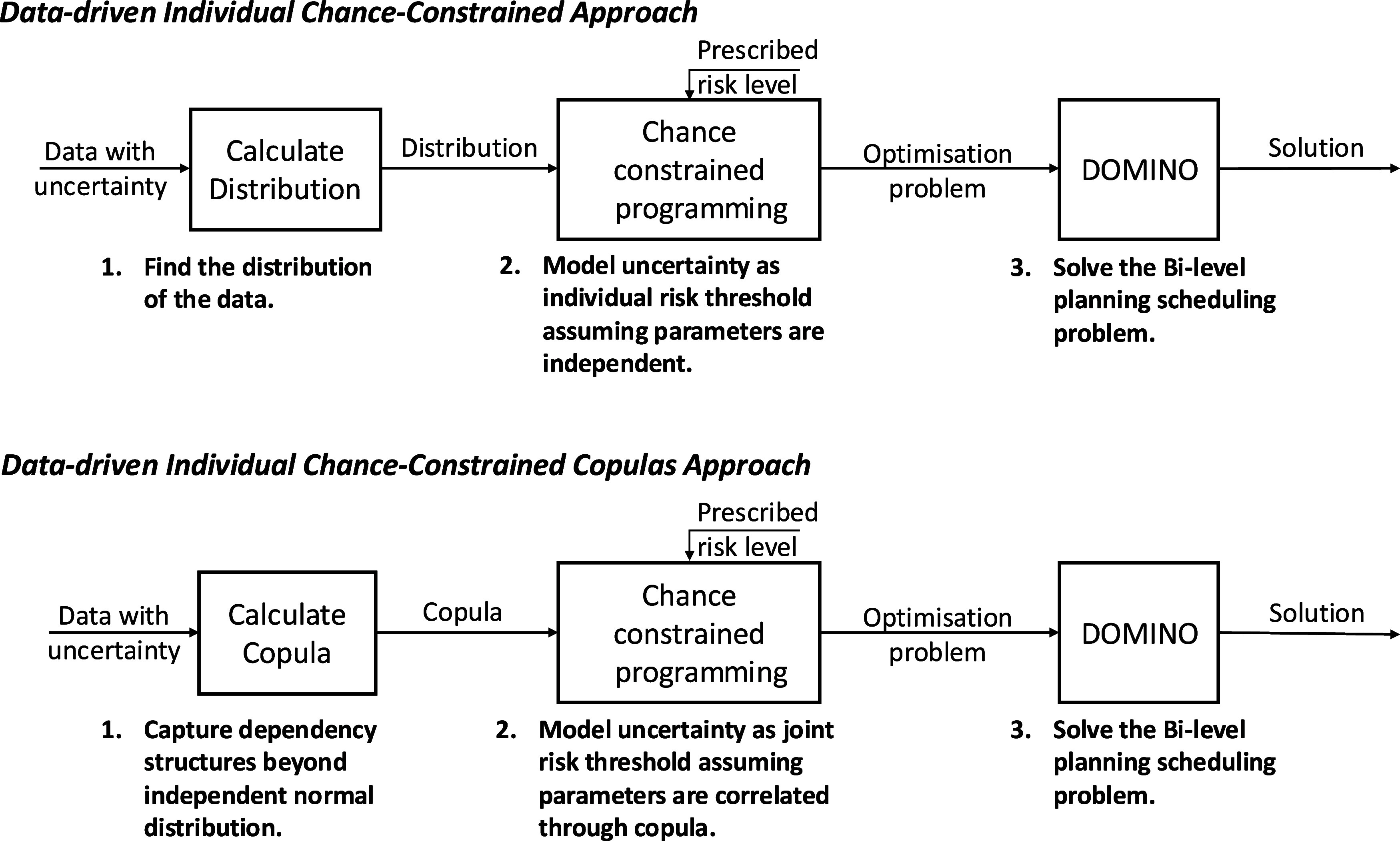
Logic flow for the proposed data-driven chance constrained framework and with copulas approach in an iPS problem context. Upper: conventional ICC model with uncertainty calculated via individual risk thresholds under full independence among uncorrelated demands. Lower: C^3^IP-DOMINO model with parameter correlations estimated by copula.

## 3 Case Studies

### 3.1 Experimental Setup

We test our proposed C^3^IP-DOMINO framework on problems from the process system engineering literature, with a particular focus on three case studies derived from the planning and scheduling of a multiproduct manufacturing process^13^ and a crude oil refinery scheduling problem.^26,39^ We employ the DOMINO framework to solve bilevel optimization problems. Specifically, the nonlinear aspects of the integrated problem stem the presence of sequence-dependent changeovers with variable times and bilinear terms arising from blending operations. The motivating example formulates CCP with an integrated copula model, and for the remaining two case studies, we further integrate the model into the DOMINO framework to solve the resulting bilevel MINLP via a data-driven approach. For the iPS problem in the context of both the multiproduct manufacturing and the crude oil refinery case studies, uncertainty arises at the planning level and is associated with demand variations across different scenarios. Moreover, for the crude oil refinery process, uncertainty also manifests at the scheduling level, where it takes the form of vessel arrival times and the minimum, maximum, and initial concentration levels of each component in each storage tank.

The specific problem and data design for the experiments are detailed below. For the motivating example and case study 1, we examine an iPS problem that incorporates demand uncertainty in a continuous multiproduct process. Specifically, we consider a continuous-time, single-stage production process carried out in a continuous stirred-tank reactor (CSTR), involving six distinct products over a two-week planning horizon.^12,39^ For case study 2, we examine the crude oil scheduling problem.^26^

Throughout the experiment, two distinct sets of realizations are systematically generated. Each realization corresponds to an independent data run generated with a fixed demand scenario, defined by a specific configuration of demand quantities across all products and periods. The first set of realizations is used to train the copula model, enabling it to estimate the empirical CDF and construct the quantile function required for the joint CCP, thereby mapping each demand quantity to an associated risk level. The second set of realizations serves as a benchmark for evaluating performance in terms of demand satisfaction rates during the Monte Carlo simulation process. For the motivating example, a set of 5000 realizations is generated to train the copulas model under the regular vine copula configuration (R-vine) and a risk level of 0.05. For both case studies, we apply a larger set containing 10,000 realizations and the same R-vine copula type with a risk level of 0.01.

For the two case studies involving DOMINO, we first define the desired risk level and use the pretrained copula model to generate the corresponding estimated uncertain demands for all products, which are input into the DOMINO framework to optimize the process of determining data-driven production level based on the input demand and evaluate the demand satisfaction rate through a simulation-based evaluation process. Within the DOMINO framework, the case study is executed 10 times on a High-Performance Computing (HPC) machine using Red Hat Enterprise Linux 8.9 (Ootpa). The NOMAD algorithm within the DOMINO framework is executed serially, utilizing one core per node with 48 GB of RAM, starting the NOMAD algorithm search from a random initial point. This point initializes the seeds of the search process using randomly generated values within the allowed bounds of decision variables, which does not necessarily need to lie within the feasible region.

For the C^3^IP-DOMINO framework computational time, we define the offline computation time to include both the construction of chance constraints and copulas fitting, whose runtimes of multiple sample sizes are evaluated in the motivating example. The offline computation also accounts for the Monte Carlo simulations. In contrast, the online computational time of running DOMINO is assessed separately in case study 1 and 2. For the case studies, we do not explicitly report the computational time for a single lower level (scheduling) solve, for it substantially depends on the selected input sample by DOMINO. The scheduling problem exhibits strong sample-dependent variability, with some instances converging significantly faster than others. Consequently, reporting a single representative scheduling solve time would not provide a meaningful measure of computational performance.

### 3.2 Motivating Example

In this motivating example, we examine an iPS problem to determine the optimal production strategy for each product in each period under uncertain demand. The problem configuration and the mixed-integer nonlinear programming (MINLP) formulation are presented in.^12^ The iPS problem is based on a production process involving 6 products and 2 planning periods (weeks), where the objective is to maximize the total profit while minimizing the penalties from inventory and backlog over the time horizon. Uncertainty is introduced in the demand from customers, which in turn influences the production target and schedule for the decision-maker. The iPS problem is formulated as an MINLP with 108 binary variables, 12 integer variables, 132 continuous variables, and a total of 280 constraints.

This motivating example aims to showcase the advantage of involving inner-data correlation using copulas, emphasizing that the copula-generated production plan as an input to DOMINO will be feasible for every possible scenario realization with a 0.01 risk level, or equivalently 99% of the time.

Among different methods,^4,7,34^ the study of the iPS through a bilevel viewpoint has been proposed. While casting the iPS problem in a bilevel fashion naturally reflects the decision-making hierarchy, the computational effort involved can be extraneous. Research on the bilevel iPS problem under uncertainty remains limited,^7^ and the case involving nonconvex lower levels is even less studied. We therefore propose a novel chance constrained-based approach. Consequently, rather than following the stochastic scenario analysis method as proposed in,^7^ which addresses every scenario in an iterative manner and can be highly resource-intensive and time-consuming, we utilize copulas to identify the multivariate dependency among product demands. We then compute the copula-estimated CDF in an offline manner, prior to the decision-making stage. This approach enables immediate, on-the-fly demand estimation during optimization and thereby substantially reducing overall computational time.

In our experimental design, the primary objective is to achieve a 99% demand satisfaction (or an equivalent risk level of 0.01) during the generated production schedules. To evaluate the effectiveness of our approach, we assess how the actual performance (a posteriori) in a stochastic environment compares to the target reliability level (a priori) initially assumed and established based on the copula-generated production target. Specifically, we begin by calculating a fixed schedule based on the copula-estimated demand level, which is then set as the production target to represent the baseline production strategy for all demand scenarios. Following this baseline, we incorporate the copula to simulate demand under varying demand conditions, generating a range of demand scenarios and systematically evaluating the frequency with which the simulated production target satisfies all predefined operational scenarios and constraints. This approach measures the robustness of copula-estimated demands in handling uncertainty in production planning.

The discrepancy between the a priori and a posteriori performances may arise, which may be attributed to the copula method being data-driven and nonparametric. Since our approach does not impose any prior assumptions regarding the data structure or parameter settings of the underlying distribution for the demand data, we instead rely on the observed data to train the best-fitting copula model to estimate the CDF of joint and individual demands. To examine the impact of this discrepancy, we conduct an experiment aimed at isolating and evaluating the extent to which the incorporated scenario size during the copula model training process affects the accuracy of predictions related to demand satisfaction. Specifically, we employ the *joint* and *individual* demand satisfaction rates as key quantitative metrics to assess the quality and reliability of copula-generated demand under a predefined risk level, as well as the consistency in how accurately the copula approach aligns with expected outcomes under different scenario sizes. The joint satisfaction rate represents the proportion of simulated scenarios in which all demand targets are simultaneously satisfied, or equivalently, the proportion of scenarios in which no backlog occurred. In contrast, the individual satisfaction rate evaluates each individual product’s demand independently, without accounting for correlations or interdependencies among them. Multiplying these separate rates yields the independently joint satisfaction level, a metric under the assumption that all demands are individually met without considering any correlation. The joint and individual satisfaction rates can be calculated using eqs 12 and 12a, respectively.
10

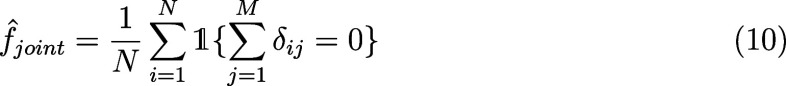



11

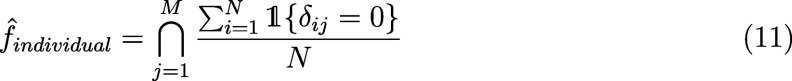

where δ_*ij*_ represents the backlog level for product index *j* in realization *i*. The indicator function 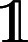 gives a binary outcome of value 1 if the condition is satisfied and 0 if not.

Figure 3 showcases the satisfaction rate and computational time required to train the copula under different historical data set sizes. Results demonstrate that the copula model, which assumes correlation among demands, constantly yields higher joint satisfaction rates than the multiplication of individual rates under independency assumption, highlighting the copula model’s capacity to capture the collective behavior of demand data. Moreover, a higher joint satisfaction rate produced by the copula suggests that interactions between demand levels may confer additional value. Incorporating these interactions could be crucial in increasing the overall satisfaction rate among all products. Furthermore, Figure 3 highlights the satisfaction rate averaged from 10 rounds with different numbers of generated scenarios. We observe a consistently high joint satisfaction rate as the total number of training scenarios increases from 1000 to 10,000. This suggests that the chance constrained copulas model has a high accuracy in estimating the CDF, resulting in the generation of a fixed production level that ultimately increases the likelihood of demand satisfaction compared to the multiplication of individual marginal satisfaction rates.

**Figure 3:**
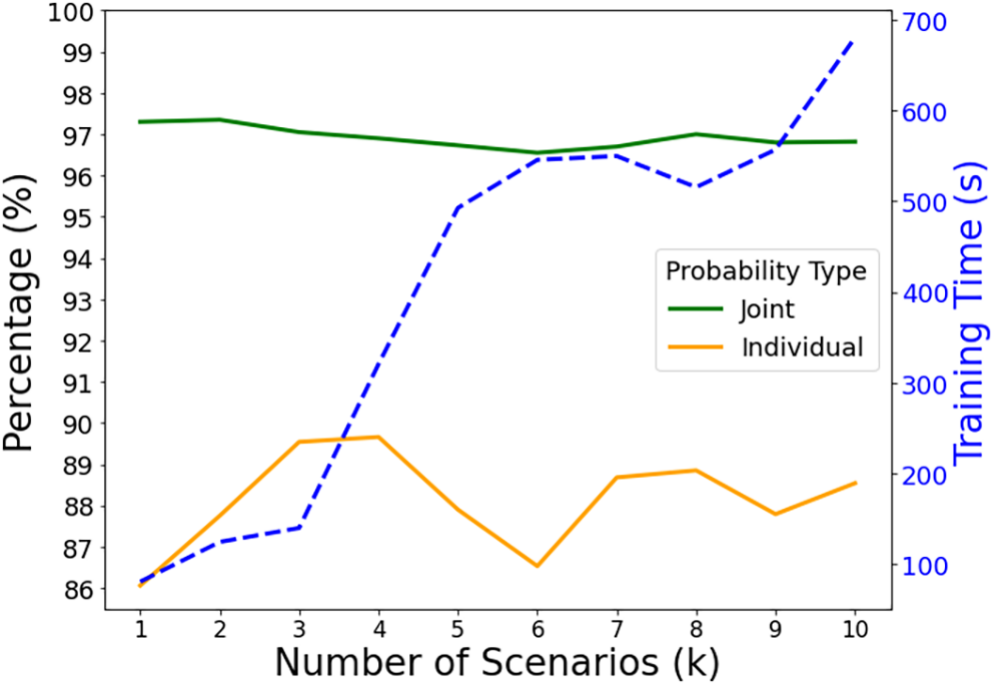
Joint and individual satisfaction rate (left *y*-axis solid lines) and copula training time (right *y*-axis dotted lines) for copula-estimated demand of instance size 6 products and 2 weeks with different numbers of generated scenarios. All results are averaged over 10 computational rounds.

The average computational time required for DOMINO to solve the integrated planning and scheduling problem is approximately 10 h for an instance size of 6 products and 2 planning periods. This underscores the significance of managing uncertainty in an offline manner, as advocated by our proposed data-driven copulas framework. Additionally, our proposed copula approach requires estimating the CDF and solving the model only once, hence further enhancing its efficiency.

We also assess the quality and robustness of the copula-estimated production sequence on all the demand scenarios compared to tackling these scenarios individually. In Figure 4a, we have an average backlog cost of 5.08 and a joint satisfaction rate of 99.9%, compared to the optimal solution created by solving the MINLP formulation with a backlog cost of 0.01 and a joint satisfaction rate of 100% in Figure 4b. Once the copula model is trained, it is used to generate a production sequence that is used for all the evaluation realizations.

**Figure 4:**
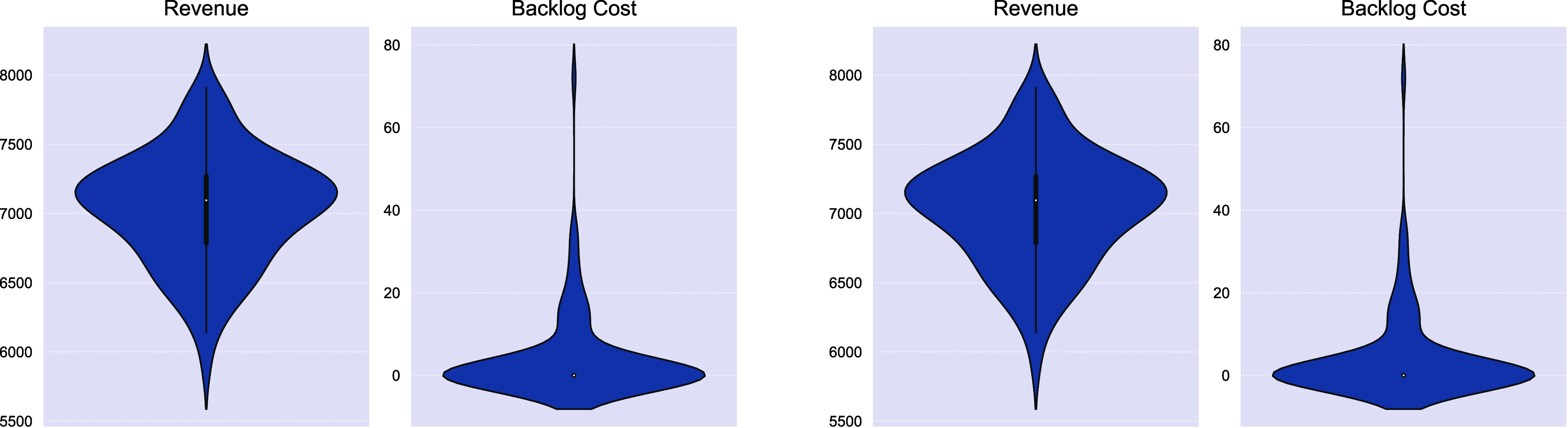
100 solutions solved via chance constrained copula framework with fixed estimated production sequence (left) and as individual problems with different production sequences (right).

### 3.3 Multiproduct Planning and Scheduling Case Study

In this case study, we continue to examine the integrated planning and scheduling problem with uncertain demand, building on the findings from the motivation study. The configuration of this case study involving 6 products, 2 planning periods (weeks) and 10,000 realizations at a risk level of 1% are presented for the chance constrained copula model C^3^IP and its deterministic equivalent, which is a conventional ICC formulation that determines the production target by simply setting it to the demand scenario corresponding to the top 99% highest total demand aggregated from 6 products and 2 weeks. The estimated demands from both C^3^IP and deterministic models are then provided individually to the DOMINO framework, where the integrated planning and scheduling problem is addressed.

After the copula-estimated demand set is input into the DOMINO framework, the NOMAD algorithm was applied to tackle the upper-level (data-driven) problem, while BARON was employed to solve the lower-level problem to global optimality. Once the demands are generated and input into DOMINO, a total of 10 runs were conducted inside DOMINO with the outcome for all runs listed in Table 1. The solution time for a single lower-level scheduling problem can be obtained by dividing the total computational time by the number of evaluated samples. Based on this calculation, the average solution time per sample round is approximately 4 s for the conventional ICC model, and 4.3 s for the proposed C^3^IP model. More specific results regarding the production target and demand level outcomes for the best scenario out of the 10 runs are presented in Figure 5. The inventory levels for all products over the 2 weeks are zero according to the result.

**Figure 5:**
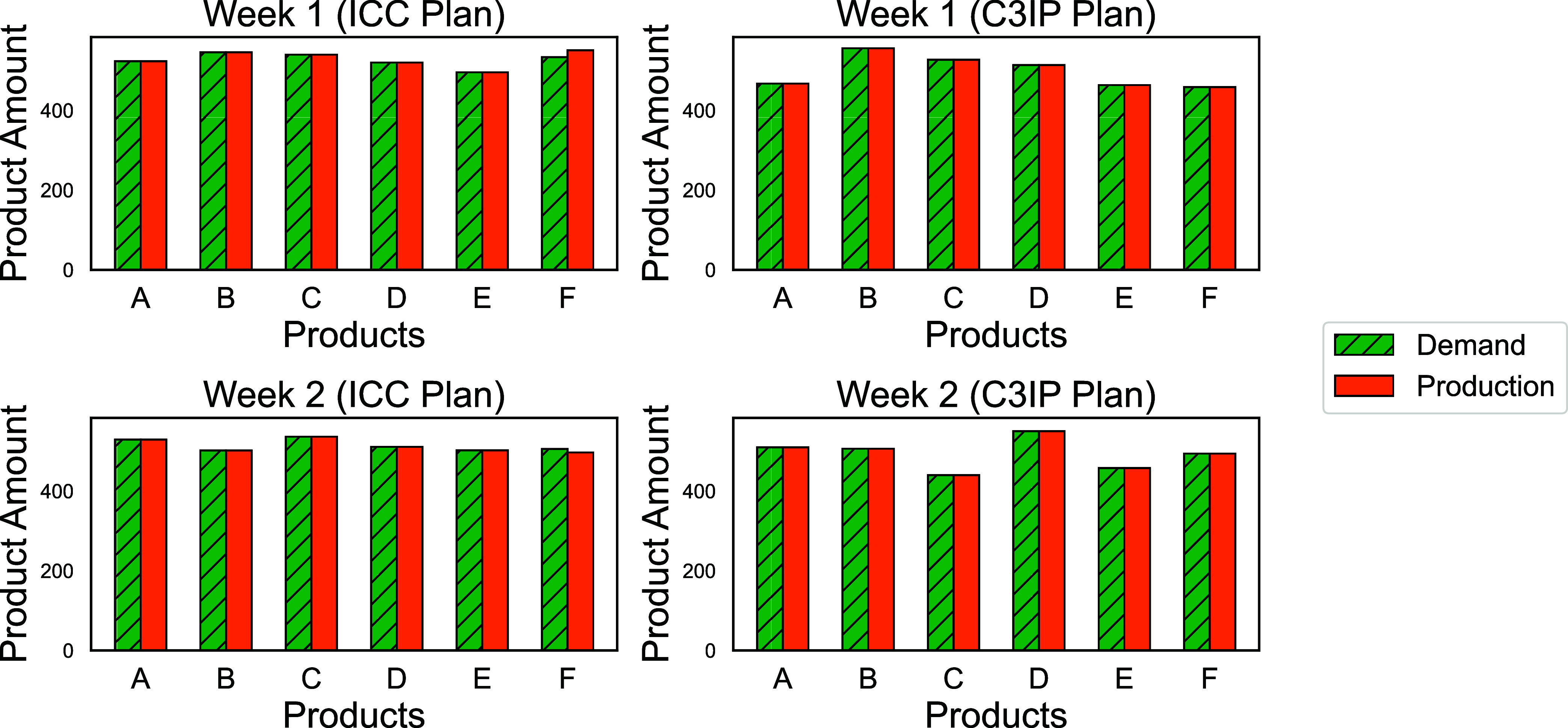
Best-found solution containing production and inventory levels solved by DOMINO for the 6-product 2-week instance with copula-generated and deterministic demand level under 10k scenarios. All inventory levels are zero and hence not shown in the plot.

**Table 1:** Performance Comparison between Chance Constrained Copula Model (**C** ^**3**^ **IP**) and Its Deterministic Equivalent as a Conventional ICC Model

	**conventional ICC model (99%)**			**C** ^**3**^ **IP model**		
runs	time (h)	profit (k)	sample	time (h)	profit (k)	sample
1	12.54	5.21	9963	9.61	4.93	8588
2	8.42	4.08	7560	10.33	4.64	8394
3	6.57	5.21	5850	10.16	4.91	8212
4	7.94	4.82	7098	8.64	4.61	6973
5	8.83	3.99	7956	15.07	4.94	13,217
6	8.50	5.05	7795	7.70	4.94	6500
7	6.86	5.21	6260	6.81	4.93	5667
8	7.75	5.21	6889	9.00	4.93	7899
9	7.55	5.21	6752	6.93	4.93	5969
10	11.27	5.21	10565	18.26	4.93	13,543
**average**	8.62	4.92	7668.80	10.25	4.87	8496.20
**STD**	1.79	0.46	1443.22	3.48	0.12	2625.04

In practice, a risk-averse way of demand estimation is generally associated with higher production targets. This is because risk aversion tends to overestimate the demand profile, which can lead to increased production levels in the event of potential backlogs. The underlying rationale suggests that by anticipating more extreme scenarios in which demand levels are significantly higher, decision-makers can reduce the risk of underproduction, avoid demand violations, and thereby increase the overall demand satisfaction rates. However, excessively overestimating the production target to safeguard against backlogs may, in turn, generate additional inventory costs. Moreover, while this strategy may improve short-term resilience for a certain subset of products, it can compromise the robustness and adaptability of the production planning process. This is particularly obvious when the total production time or resource budget is limited. Such limitations become particularly evident when the time budget is tightly constrained, as certain product types may not be produced in time due to the overproduction of other products.

From Table 1, the deterministic demand case currently yields a higher average production target, therefore higher average sales profit, than the copula-generated uncertainty case with a 99% confidence level. The proposed C^3^IP model, characterized as a risk-averse approach, may be associated with a slightly reduced profit nominally due to being risk-averse in production targets and sales. Nonetheless, the model is specifically designed to capture the correlations between product demands and to generate more realistic and balanced production targets, thereby mitigating the risk of overproduction and the associated increase in inventory costs. As a result, the copula-generated demands tend to be lower than their deterministic counterparts, reflecting a closer and more accurate representation of the demand profile. The results shown in Table 1 indicate that although the copula-estimated demand is lower, which contributes to a lower objective value compared to that from the deterministic model estimation with a 99% confidence level, the difference in profit is only marginal. Additionally, we observe that copula-generated demands lead to smaller variability in objective value, suggesting a more stable estimation in the demand profile throughout the different runs compared to the deterministic counterpart. Therefore, the integration of the copula technique contributes to greater robustness and its practicality for real-world applications.

Next, we evaluate the performance of the two models through a Monte Carlo simulation to investigate how the robustness of our proposed C^3^IP model can mitigate the overestimation of demand, thereby reducing the penalty while maintaining a promising level of profit. The Monte Carlo simulation begins with the generation of a set of sample scenarios, each based on the assumption of a multivariate normal distribution for all demands. Specifically, 5000 scenarios are utilized to train the C^3^IP model. This provides stable estimation of both the copula dependence structure and the tail quantiles used in the chance constraints. The training procedure follows the specific steps illustrated in Figure 1, which guide the selection and tuning of the copula family and parameters to fit the model across scenarios and ensure model robustness. Based on the generated training scenarios, the proposed C^3^IP model constructs a 95% quantile estimated demand set that satisfies 95% confidence level of the anticipated demand for all products over a period of 2 weeks. In addition, we generate demands for comparison with the conventional ICC formulation, referred to as the “deterministic model”, where the production target is determined by selecting the demand scenario corresponding to the α-percentile of total demand aggregated over all products and periods. The demands are generated for each target confidence level, including thresholds of 90%, 95% and 99%. This is to enable a comparative evaluation of the performance of different deterministic models using similar criteria for production satisfaction rates. We refer to the chance constrained 95%-quantile C^3^IP model as C3IP and denote the deterministic models as ICC90, ICC95 and ICC99 corresponding to their respective predefined confidence levels. Next, the demands estimated by each model are input into the planning and scheduling optimization problem as proposed in^12^ and solved as a MINLP formulation to reach a solution. For each estimated demand, the corresponding MINLP formulation is solved to optimality to evaluate the objective function. We obtain the dry-run key performance metrics, including expected profit, expected inventory and the expected level of backlog from the solution. These performance metrics are used to quantify the overall profit and penalties associated with a specific model-estimated demand set, thereby evaluating the performance of a certain model. Ideally, the demand estimation from each model should be representative in capturing the characteristics of a wide range of potential scenarios, enabling robustness to consistently maintain high overall profit when evaluated against actual demand realizations from scenarios seen beyond the training data. Specifically, we generate another sample of evaluation scenarios that are independent of the training set, with multivariate-distributed product demands. A sample size of 2000 scenarios is determined in order to ensure reliable sample average convergence. The MINLP formulation is solved once for each estimated demand instance generated by the copula and deterministic models. The expected total profit, inventory and backlog from the MINLP formulation are accordingly stored as the dotted lines in Figure 6 corresponding to each model’s demand. Additionally, the decision variables corresponding to the production target and time scheduling are stored. These two sets of variables are then fixed and reused across the evaluation scenarios to conduct a performance comparison between the copula and deterministic models. The total profit, inventory, and backlog levels are computed for each realization. In Figure 6, the solid lines reflect the actual demand realization for each realization and illustrate how the results evolve as more scenarios are considered. The results are generated using the following formula:

12 $$\begin{array}{l} {\mathrm{CumProf}}^{\left(i\right)}={\mathrm{CumProf}}^{\left(i-1\right)}+{\mathrm{Prof}}^{\left(i\right)}\\ {\mathrm{EV}}^{\left(i\right)}=\frac{{\mathrm{CumProf}}^{\left(i\right)}}{N_{i}} \end{array}$$

where Prof^(*i*)^ represents the profit computed from realization *i*, while CumProf^(*i*)^ denotes the total cumulative profit from all the previous realizations. The expected value EV is the total cumulative profit divided by the number of realizations *N*_*i*_ considered for the cumulation, where 2 ≤ *i* ≤ *N* and 1 ≤ *N*_*i*_ ≤ *N*. The expected values for the backlog and inventory penalties are computed using the same approach based on eq 14, in which the cumulative profit and iterative profit are replaced by the same pair for the backlog and inventory. For each realization, the objective value is computed as the sum of profit minus the penalties for backlog and inventory. To evaluate all models’ prediction accuracy, we track the difference between the dry-run and EV for all models by comparing the gap between the objective values derived from MILP model based on estimated demands with the actual realized demand from each realization.

**Figure 6:**
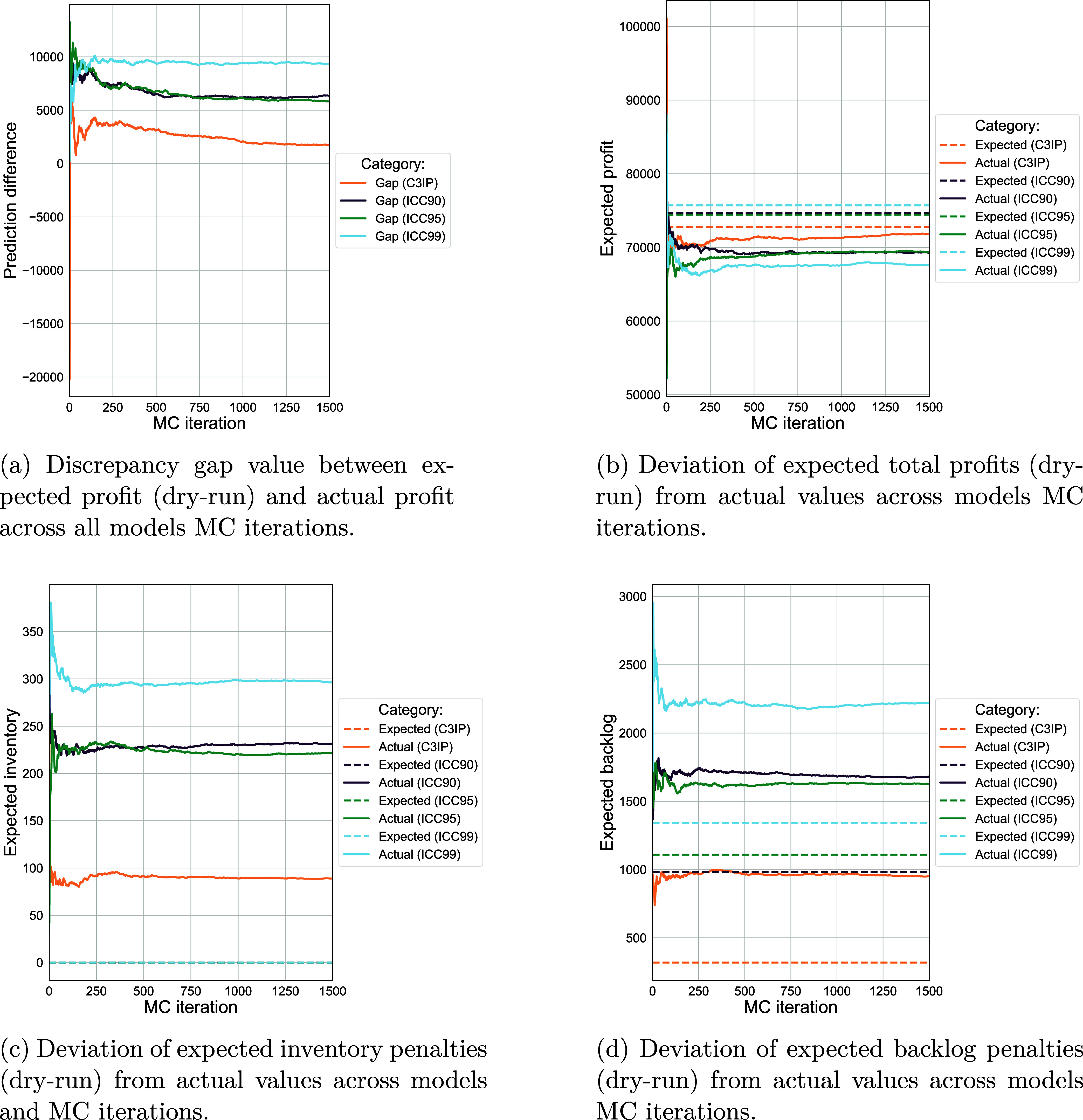
Monte Carlo simulation for the dry-run MINLP model (dry-run) and the reduced model (EV) with known estimated production target and scheduling decisions. The plots illustrate the production profit expected-actual gap (top left), profit (top right), inventory (bottom left), and backlog (bottom right) between 95%-quantile C^3^IP model and 90, 95, 99% for the deterministic model. The higher the better for the two profit plots, the lower the better for the inventory and backlog penality plots. Graphs show the first 1500 scenarios when convergence achieved.

We observe in Figure 6 that the solid lines plateau in all the plots, showing that the performance converges for both copula and deterministic models when sufficient evaluation scenarios are considered. By subtracting the realized values generated from the Monte Carlo simulation from the expected values, we obtain the difference between anticipated and actual outcomes in objective value function. This discrepancy can be visualized in Figure 6a to highlight the performance gap. The plot reveals that the C^3^IP model produces a much smaller gap between the dry-run expected value (dotted lines) and the actual realization of profit (solid lines) across the evaluation scenarios, demonstrating a more accurate prediction of the demand compared to the deterministic models, even the one that achieves higher confidence level of 99%. Additionally, the copula-estimated expected production target typically yields a lower value of aggregated demand compared to its deterministic equivalent targets, primarily due to the exploitation of interdata correlation. Because of this advantage, the C^3^IP model produces much smaller inventory levels, thus incurs lower inventory costs, compared to its deterministic counterparts, as observed from Figure 6c. These expected production targets generated by the deterministic models under high confidence levels are more likely to lead to overproduction, as the actual demands under most scenarios can be sufficiently met, thereby creating higher inventory costs.

From Figure 6d, the copula-estimated production target yields the smallest expected backlog level across all evaluation scenarios. This outcome can be attributed to the limitations inherent in deterministic models. For a deterministic model with a lower confidence level (ICC90), the estimated production target may be insufficient to meet the actual demands of most scenarios, resulting in a significant backlog level. On the contrary, a deterministic model with a higher confidence level (ICC99) incurs a higher estimated production target but also a higher expected backlog due to the inefficiencies arising from a fixed production time capacity per period that may not adequately support the full range of production in a timely manner.

Overall, the C^3^IP model demonstrates greater robustness and provides a reliable estimate of demands, generating higher average profits and lower penalties in terms of backlog and inventory. Moreover, the C^3^IP model produces the smallest discrepancy in objective value between the expected and actual demand realization across all scenarios.

### 3.4 Crude Oil Planning and Scheduling Case Study

In this case study, we examine a refinery scheduling problem comprising vessels for unloading, storage tanks, charging tanks, and crude oil distillation units (CDUs), as illustrated in Figure 7. Full details of the system configuration are provided in our previous work.^40^ The objective of the scheduling problem is to minimize the total cost of production, and the planning levels objective function is to minimize the cost over the entire time horizon.

**Figure 7:**
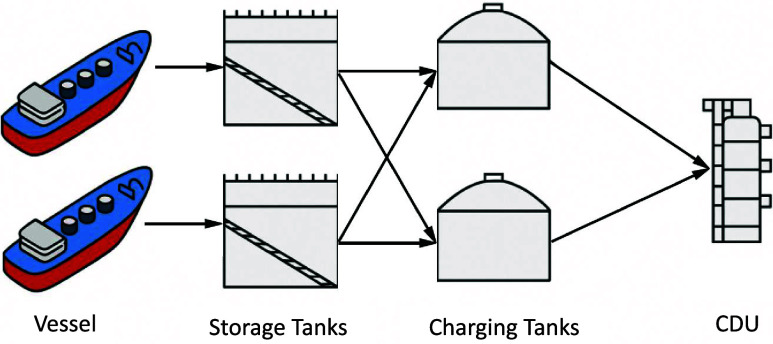
Configuration of the crude oil unloading and refining network. Adapted from ref 40. Reproduced from ref 40 © Author(s) 2025. This article is licensed under a Creative Commons Attribution (CC BY) license.

This example involves the operation of the system during a 15-day period with two crude oil products. Uncertainty is introduced in several critical parameters, including: (i) demand for each product, (ii) vessel arrival times, (iii) minimum, maximum, and initial concentrations of component *k* in the crude oil of each storage tank (iv) initial concentration of component *k* in the crude-oil mix of each charging tank, (v) upper and lower concentration limits of component *k* in charging tanks, and (vi) the initial volume of the crude-oil mix in each charging tank.

We select a list of parameters from the crude oil refinery operations data in ref 40 and generate the uncertainty version for the framework using the multivariate normal distribution with standard deviation being 10% of their corresponding mean value. A detailed list of these uncertain parameters, as well as their corresponding mean values, is provided in Table 2. These uncertain parameters are generated utilizing the copula framework and are sampled and input into the DOMINO framework to assess resilience and performance. The upper-level (planning problem) has 28 decision variables and 61 nonlinear gray-box constraints. The scheduling problem comprises 24 binary variables and 134 continuous variables, with a total of 386 constraints. We solve the lower-level MINLP with BARON, while the comprehensive bilevel structure is optimized utilizing the NOMAD solver within the DOMINO framework. This integration facilitates effective management of uncertainty by merging copula-based chance constrained estimation with the DOMINO framework in both planning and scheduling levels.

**Table 2:** Uncertain Parameters for Crude Oil Scheduling Problem in Case Study 2^a^

**parameter**	**description**	**mean value**
demand	crude oil demand for each product per day	see Table C2
*Tarr* (*v*)	arrival time of vessel *v*	*v*_1_ = 1, *v*_2_ = 5
*Dsmin* (*i*,*k*)	minimum concentration of component *k* in crude oil of storage tank *i*	*i*_1_ = 0.01, *i*_2_ = 0.06
*Dsmax* (*i*,*k*)	maximum concentration of component *k* in crude oil of storage tank *i*	*i*_1_ = 0.02, *i*_2_ = 0.07
Vv0 (*v*)	initial amount of crude oil in vessel *v*	*v*_1_ = 100, *v*_2_ = 100
Dv (*v*,*k*)	concentration of component *k* in crude oil of vessel *v*	*v*_1_ = 0.01, *v*_2_ = 0.06
*Dbmin* (*j*,*k*)	minimum concentration of component *k* in crude oil mixture for charging tank *j*	*j*_1_ = 0.015, *j*_2_ = 0.045
*Dbmax* (*j*,*k*)	maximum concentration of component *k* in crude oil mixture for charging tank *j*	*j*_1_ = 0.025, *j*_2_ = 0.055

a The production follows Figure 7, including 2 vessels (*v*), 2 storage tanks (*i*) and 2 charging tanks (*j*). The mean values for each parameters are taken from numerical settings for example 1 in ref 40, while the standard deviation is set to 10% of the corresponding mean. For instance, *v*_2_ = 5 for *Tarr*(*v*) parameter indicates that the mean arrival time of vessel 2 is 5 with a standard deviation of 0.5 for multivariate distribution value generation.

We use the CCP model to generate uncertain parameters using a multivariate distribution with lower and upper bounds given in the table below. We consider 10,000 scenarios and a risk level of 0.01. The C^3^IP-DOMINO framework is run 10 times to generate both the objective value and the sample size. We give an upper computational time limit of 144 h on the HPC. The results are illustrated in Table 3 and Figure 8. In Table 3, the objective function for the bilevel problem at the lower scheduling level is to minimize production cost, and at the upper planning level, it is to minimize planning cost over the entire time horizon. The reported objective values are the upper-level planning costs, which contain the scheduling-level objective (production cost). The sample refers to the number of trial points generated and evaluated during the optimization process. The corresponding per-round solution times are 157.2 s for the conventional ICC model and 168.5 s for the C^3^IP model.

**Figure 8:**
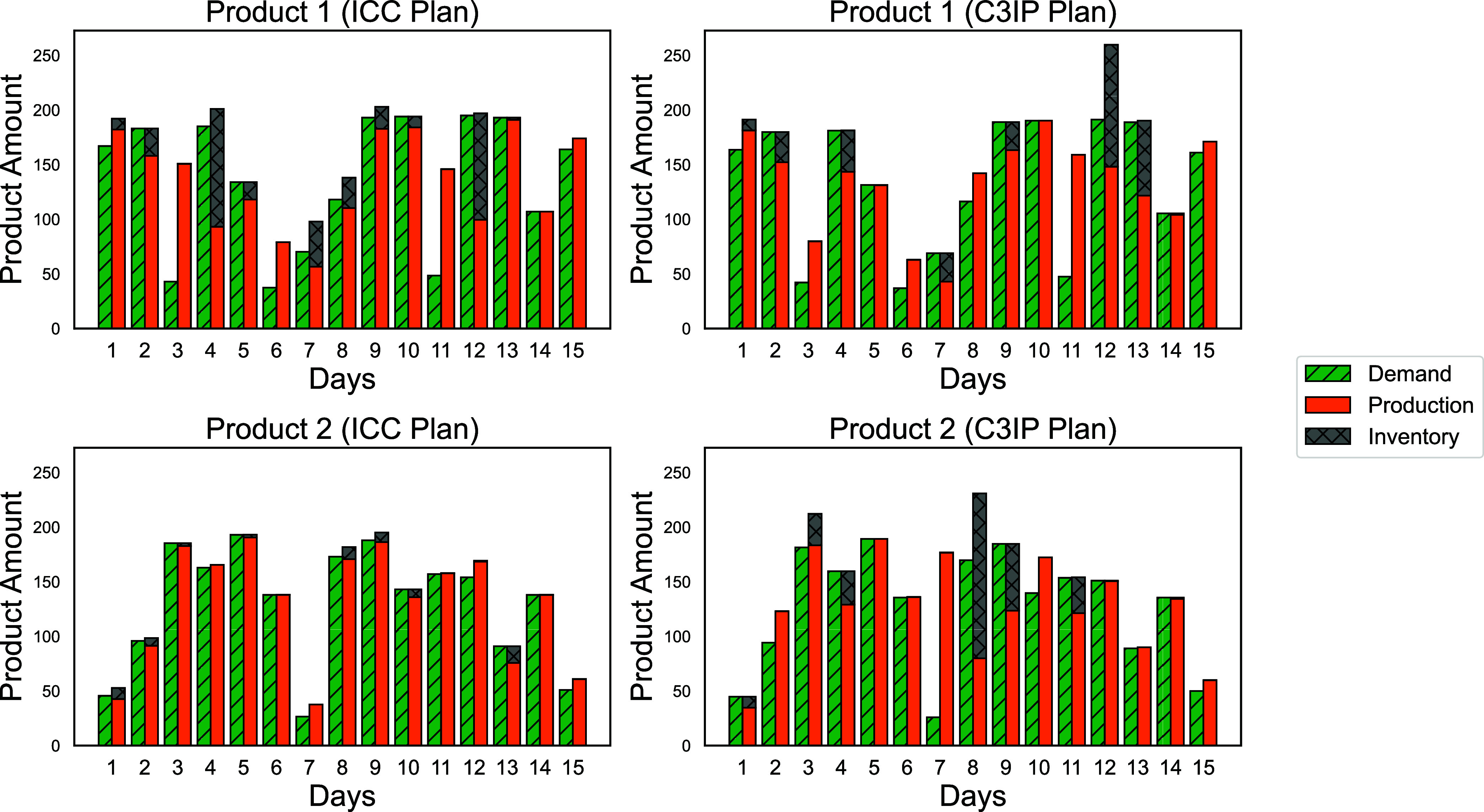
Best-found solution containing production and inventory levels solved by DOMINO for the 2-product 15-day instance with copula-generated and deterministic demand level under 10k scenarios.

**Table 3:** Performance Comparison between Chance Constrained C^3^IP Model and its 99%-Quantile Deterministic Equivalent Using ICC^a^

	**conventional ICC Model (99%)**			**C** ^**3**^ **IP Model**		
runs	time (h)	objective (k)	sample	time (h)	objective (k)	sample
1	144	32.69	3047	144	57.11	1645
2	144	58.58	1676	144	50.77	3184
3	144	44.20	6534	144	72.60	1853
4	144	81.98	801	144	93.83	1034
5	144	94.80	103	144	33.97	5934
6	144	23.85	2867	144	50.77	1283
7	144	58.63	6189	144	138.09	96
8	144	50.72	2960	144	69.96	1871
9	144	46.43	3777	144	41.41	8677
10	144	45.27	5061	144	34.96	5195
**average**	144	53.71	3301.50	144	64.35	3077.20
**STD**	0	20.23	2039.71	0	30.24	2555.42

a Objective is planning cost, lower the better.

Overall, the predicted demand level is lower under the CCP plan compared to the deterministic plan, which corresponds to a lower production level for both types of products under the CCP plan. Specifically, we observe an outlier under the chance constrained C^3^IP model result in Run 7. This suboptimal solution is due to only a limited number of 96 samples being drawn, and the searching process was terminated by GAMS due to DOMINO initialization issues, hence reaching the full time limit of 144 h. A similar pattern is observed for Run 5, in which the 99%-quantile deterministic equivalent model has DOMINO returning a solution with much higher planning costs than the chance constrained C^3^IP model. Furthermore, the deterministic data-driven formulation outperforms scenario-based approaches, as the latter exacerbates the search space for the data-driven optimizer. We notice that the crude oil problem is generally more challenging to tackle than the planning and scheduling problem in Case Study 1, and is more sensitive to the initialization of the search in DOMINO.

In Figure 8, we observe a higher production level on days 3 and 11 for product 1 under the deterministic plan, which leads to a surge in the inventory level on days 4 and 11. On the contrary, under the CCP plan, the production exhibits a higher level only on day 10, resulting in a peak in the inventory level on day 11. Additionally, the demand level on day 6 is predicted to be lower under the CCP plan compared to the deterministic plan, whereas the demand for day 7 is predicted to be higher. This results in a higher inventory level under the CCP plan on day 7.

## 4 Conclusions

We present a chance constrained optimization framework with copula functions to effectively capture and model the multivariate demand data dependency structure with applications to integrated planning and scheduling problems. Our approach ensures that decision-making is feasible within a predefined risk threshold. We validated the copulas-generated production targets using the DOMINO framework to solve the resulting data-driven bilevel MINLPs. Our computational experiments demonstrate that the proposed approach is capable of identifying robust solutions that result in higher joint satisfaction rates than the conventional individual chance constraint formulation for products and near-optimal performance, all while keeping reasonable computational time.

## A—Bilevel iPS Formulation

We showcase the mathematically formulation for two bilevel iPS problems in the integrated planning and scheduling of manufacturing and crude oil refinery as follows.

The integrated planning and scheduling manufacturing baseline formulation is introduced in ref 12, where the production target is estimated during the planning level, and the scheduling is determined using constraints (8)-(25) as proposed in the paper. The production target is estimated via

A1
$$P\left(x_{i}\geq D_{i}\right)=1-\alpha_{i},\forall i\in \left\{1,...,n\right\}$$

where for each production constraint *i*, the corresponding production target *x*_*i*_ must satisfy the associated uncertain demand *D*_*i*_, such that the specified demand requirement constraint is fulfilled with a probability no less than (1−α_*i*_)% for each individual scenario or realization.

The crude oil refinery baseline formulation is introduced in ref 40 as the instance “Crude Oil Refinery Operations”. The source of uncertainties at the planning level includes the production target shown as the crude oil demand. Uncertainties in the scheduling level include the vessel arrival times, the minimum, maximum, and initial concentrations of each component in the crude oil of each storage. We apply the following replacement to substitute the deterministic constraints in the baseline iPS formulation to place the chance constraints.

For the planning level, the original demand level constraint as Con(40) in ref 40 for tracking the demand *Pr* is replaced by

$$P\left(Pr_{j}\leq \sum_{l}\sum_{n}B_{j\ln}\right)\geq 1-\alpha ,\forall j\in J$$
A2

$$P\left(Pr_{j}\geq \sum_{l}\sum_{n}B_{j\ln}\right)\geq 1-\alpha ,\forall j\in J$$
A3

The original material balance constraints as Con(1) and Con(2) in ref 40 for tracking Vv0_v_ for the initial amount of crude oil in vessel *v* are replaced by

A4
$$P\left(\mathrm{Vv}0_{v}\leq \sum_{i\in I_{v}\;}\sum_{n}Bv_{v,i,n}\right)\geq 1-\alpha ,\forall v\in V$$

A5
$$P\left(\mathrm{Vv}0_{v}\geq \sum_{i\in I_{v}\;}\sum_{n}Bv_{v,i,n}\right)\geq 1-\alpha ,\forall v\in V$$

A6
$$P\left(\mathrm{Vv}0_{v}\geq Vv_{v,n}+\sum_{i\in I_{v}\;}\sum_{n}Bv_{v,i,n}\right)\geq 1-\alpha ,\forall v\in V,n=1$$

A7
$$P\left(\mathrm{Vv}0_{v}\leq Vv_{v,n}-\sum_{i\in I_{v}\;}\sum_{n}Bv_{v,i,n}\right)\geq 1-\alpha ,\forall v\in V,n=1$$

For the scheduling level, the original material balance constraints as Con(10) and Con(11) in ref 40 for the minimum and maximum uncertain concentrations *Dsmin* and *Dsmax* tracking the components inside storage tanks are replaced by

A8
$$P\left(\mathrm{Dsmi}n_{i,k}\leq \frac{\mathrm{Vs}s_{i,k,n}}{Vs_{i,n}}\right)\geq 1-\alpha ,\forall i\in I,\;\forall k\in K,\;\forall n\in N$$

A9
$$P\left(\mathrm{Dsma}x_{i,k}\geq \frac{\mathrm{Vs}s_{i,k,n}}{Vs_{i,n}}\right)\geq 1-\alpha ,\forall i\in I,\;\forall k\in K,\;\forall n\in N$$

The original material balance constraints as Con(16) to Con(19) in ref 40, the minimum and maximum uncertain concentrations *Dbmin* and *Dbmax* for tracking the components inside storage tanks are replaced by

A10
$$P\left(\mathrm{Dbmi}n_{j,k}\leq \frac{\mathrm{Vb}b_{j,k,n}}{Vb_{j,n}}\right)\geq 1-\alpha ,\forall j\in J,\;\forall k\in K,\;\forall n\in N$$

A11
$$P\left(\mathrm{Dbma}x_{j,k}\geq \frac{\mathrm{Vb}b_{j,k,n}}{Vb_{j,n}}\right)\geq 1-\alpha ,\forall j\in J,\;\forall k\in K,\;\forall n\in N$$

$$P\left(\mathrm{Dbmi}n_{j,k}\leq \frac{\mathrm{Bb}b_{j,l,k,n}}{Bb_{j,n}}\right)\geq 1-\alpha ,\forall j\in J,\forall l\in L,\forall k\in K,\forall n\in N$$
A12

A13
$$P\left(\mathrm{Dbma}x_{j,k}\geq \frac{\mathrm{Bb}b_{j,l,k,n}}{Bb_{j,n}}\right)\geq 1-\alpha ,\forall j\in J,\;\forall l\in L,\;\forall k\in K,\;\forall n\in N$$

The original sequence constraint as Con(41) in ref 40 for tracking the vessel to storage tank arrival time flow is replaced by

$$P\left(\mathrm{Tar}r_{v}\leq \frac{\mathrm{Tv}s_{v,i,n}}{x_{v,i,n}}\right)\geq 1-\alpha ,\forall v\in V,\;\forall i\in I_{v},\;\forall n\in N$$
A14

## B—Copula Model Comparison

We investigate different copula model structures and evaluate their performance on satisfaction rate and computational time. The experiments use an instance size of 5000 samples, consistent with Case Study 1 with 6 products and 2 weeks. Under the multivariate vine copula setting, the default copula family selection allows the copula model to automatically select bivariate copula as building blocks based on maximum likelihood estimation. Alternatively, all pair-copula blocks can be constrained to belong to a single family (e.g., only independence, only Student-t, only Clayton).

Comparative experiments using a specified single bivariate copula are reported in each row, with each configuration repeated ten times and the results averaged. The results in Table [Table tblB1] indicate that permitting a best-fit copula family for each variable pair, rather than restricting the model to a single copula family, contributes to a higher satisfaction rate on average. Although Student-t attains a higher satisfaction rate, its associated computational costs make it less preferable in practice.

**B1 tblB1:** Comparison of Copula Families Grouped by Master Structure

**structure**	**copula family**	**satisfaction rate**	**time (s)**
**R-Vine**	automatic	0.9893	2.52
	clayton	0.9695	1.38
	gumbel	0.9822	1.98
	student-t	0.9960	177.46
	indep	0.8887	0.39
	joe	0.9491	1.89
**C-Vine**	automatic	0.9863	2.72
	clayton	0.9651	1.48
	gumbel	0.9810	2.10
	student-t	0.9941	167.26
	indep	0.8869	0.31
	joe	0.9491	1.62
**D-Vine**	automatic	0.9860	2.24
	clayton	0.9675	1.31
	gumbel	0.9834	1.89
	student-t	0.9945	148.71
	indep	0.8865	0.29
	joe	0.9507	1.60

The selection of R-vine is motivated by the absence of a clear data structure in the data set, rendering specific structure assumptions unnecessary. Here we compare the R-Vine copula structure with arbitrary dependencies with C-vine, which contains a main dominant variable, and D-vine, which contains sequential neighbor dependence. The results indicate that the R-Vine structure attains the highest satisfaction rate among the evaluated alternatives.

## C—Crude Oil Case Study Demand

The crude oil demand parameter values in Case Study 2 are shown in Table [Table tblC2] with a 15-day period and two crude oil products. The demand scenarios are generated using these values as the average for the multivariate normal distribution.

**C2 tblC2:** Case Study 2 Crude Oil Demands

**product 1**	**product 2**
166.65	45.54
183.04	95.92
42.86	184.83
184.41	162.60
133.82	192.71
37.56	138.03
70.13	26.43
118.44	172.84
192.35	188.12
193.68	142.17
48.37	156.39
194.71	153.76
192.29	90.60
107.37	137.99
164.05	50.81
